# Zero problems with compositional data of physical behaviors: a comparison of three zero replacement methods

**DOI:** 10.1186/s12966-020-01029-z

**Published:** 2020-10-06

**Authors:** Charlotte Lund Rasmussen, Javier Palarea-Albaladejo, Melker Staffan Johansson, Patrick Crowley, Matthew Leigh Stevens, Nidhi Gupta, Kristina Karstad, Andreas Holtermann

**Affiliations:** 1 National Research Centre for the Working Environment, Lersø parkalle 105, 2100 Copenhagen, Denmark; 2 Department of Public Health, Section of Social Medicine, University of Copenhagen, 2100 Copenhagen, Denmark; 3 Biomathematics and Statistics Scotland, Edinburgh, EH9 3FD UK; 4 Department of Sports Science and Clinical Biomechanics, University of Southern Denmark, Odense, Denmark

**Keywords:** Physical activity, Sedentary time, Compositional data analysis, Missing data, Time-use

## Abstract

**Background:**

Researchers applying compositional data analysis to time-use data (e.g., time spent in physical behaviors) often face the problem of zeros, that is, recordings of zero time spent in any of the studied behaviors. Zeros hinder the application of compositional data analysis because the analysis is based on log-ratios. One way to overcome this challenge is to replace the zeros with sensible small values. The aim of this study was to compare the performance of three existing replacement methods used within physical behavior time-use epidemiology: simple replacement, multiplicative replacement, and log-ratio expectation-maximization (lrEM) algorithm. Moreover, we assessed the consequence of choosing replacement values higher than the lowest observed value for a given behavior.

**Method:**

Using a complete dataset based on accelerometer data from 1310 Danish adults as reference, multiple datasets were simulated across six scenarios of zeros (5–30% zeros in 5% increments). Moreover, four examples were produced based on real data, in which, 10 and 20% zeros were imposed and replaced using a replacement value of 0.5 min, 65% of the observation threshold, or an estimated value below the observation threshold. For the simulation study and the examples, the zeros were replaced using the three replacement methods and the degree of distortion introduced was assessed by comparison with the complete dataset.

**Results:**

The lrEM method outperformed the other replacement methods as it had the smallest influence on the structure of relative variation of the datasets. Both the simple and multiplicative replacements introduced higher distortion, particularly in scenarios with more than 10% zeros; although the latter, like the lrEM, does preserve the ratios between behaviors with no zeros. The examples revealed that replacing zeros with a value higher than the observation threshold severely affected the structure of relative variation.

**Conclusions:**

Given our findings, we encourage the use of replacement methods that preserve the relative structure of physical behavior data, as achieved by the multiplicative and lrEM replacements, and *to avoid* simple replacement. Moreover, we do not recommend replacing zeros with values higher than the lowest observed value for a behavior.

## Background

The amount of time spent in different physical behaviors (i.e. physical activity types, postures and sleep) is important for health [1]. An increased awareness of the co-dependency between time spent in physical behaviors has resulted in a shift in methods used for the analyses of such time-use data [2, 3]. Compositional data analysis (CoDA) accounts for this co-dependency between physical behaviors and is thus recommended for analyzing this type of time-use data [2, 4, 5]. CoDA addresses the fact that time-use data is relative*,* and thus is based on log-ratios between the time spent in each physical behavior. However, the presence of zeros hampers the application of CoDA as log-ratios that include a zero cannot be computed. Specifically, within physical behavior time-use epidemiology, we identified 15 out of 65 studies that reported zeros in their dataset (see Additional file 1 for systematic literature search), suggesting that zeros is a frequently occurring problem in physical behavior data.

One of the most frequent type of zeros encountered in the analysis of compositional data are “rounded zeros” [6]. Rounded zeros are a particular case of a not-missing-at-random (NMAR) [7], where the observed zero value is not a true zero, but refers to a rounded-off small value or an actual value which falls below a certain threshold (e.g. a detection limit of the measuring device) [4, 6]. In time-use data, a common cause of rounded zeros is the limited period of observation. That is, if the individual had been observed for a longer period, it is likely that positive values would have been registered for a given activity. For physical behaviors, an example could be the time spent in a moderate-to-vigorous intensity physical activity, such as running or stair climbing, which may not occur very often and therefore may go unobserved. Hence, we refer to observation thresholds in this context.

Three potential solutions to deal with rounded zeros are: 1) a meaningful merging of a physical behavior containing zeros with another behavior in the composition (e.g. combining running and stair climbing into moderate-to-vigorous physical activity); 2) excluding individuals with zeros; and 3) replacing the zeros with a sensible value to produce a complete dataset. Although easy to implement, combining behaviors beyond a certain level of aggregation can result in a significant loss of detail, since physical behaviors that account for a very small proportion of daily time may still have a strong impact on health (e.g. running or stair climbing) [8, 9]. Similarly, excluding individuals with zeros from the analyses is also undesirable as it is likely to introduce a selection bias and loss of statistical power. This leaves the replacement of zeros by small meaningful values as an attractive approach. However, data imputation will inevitably introduce some distortion [6, 10] and, hence, must be used with care to minimize bias and any subsequent impact on the results.

Several approaches have been developed to address the “zero problem” in compositional data in different contexts [6]. Within physical behavior time-use epidemiology, the three main replacement methods are the simple, multiplicative, and log-ratio Expectation-Maximization (lrEM) methods (see Additional file 1 for systematic literature search). Moreover, studies applying simple replacement report different values to replace zeros (e.g. 1 min or 0.5 min). The performance of these methods in preserving the structure of relative variation in the dataset has been assessed in other contexts [11–14]. However, no study has yet compared these methods nor assessed the importance of which replacement value is chosen in the context of physical behavior data. Given that these methods can have a considerable effect on the results of subsequent statistical analyses, we consider that such insights are essential for the practical application of CoDA methods in physical behavior time-use epidemiology. Accordingly, the objective of this study was 1) to conduct a formal comparison of the performance of the three zero replacement methods (i.e., simple, multiplicative and lrEM) for accelerometer data of daily time spent in physical activities, sedentary behavior and bed, and 2) to highlight the potential consequences of using an inappropriate replacement value (e.g. a value higher than the lowest observed value for a behavior).

## Methods

### Study population and data collection

This study was based on accelerometer data from three workplace-based cohorts; the Danish Physical ACTivity cohort with Objective measurements (DPhacto) [15], the New Method for Objective Measurements of Physical Activity in Daily Living (NOMAD) study [16] and the Danish Observational Study of Eldercare work and musculoskeletal disorderS (DOSES) [17]. Data collection and procedures have been described previously [15–17]. In short, eligible workers were asked to wear accelerometers for a minimum of two consecutive workdays and to complete a diary reporting time at work, time in bed and non-wear time. Daily time spent in physical activities, sedentary behaviors and time in bed were collected using data from one tri-axial ActiGraph GT3X+ accelerometer (Actigraph, Florida, U.S.A) placed on the right thigh using double-sided adhesive tape (3 M, Hair-Set, St. Paul, Minnesota, USA) and Fixomull (Fixomull BSN medical GmbH, Hamburg, Germany). The procedures for accelerometer data collection were identical across all three studies which enabled us to merge the data.

### Processing of accelerometer data

Accelerometer data, sampled at 30 Hz, were downloaded using ActiLife Software version 5.5 [18] and filtered using a 4th order Butterworth filter with a 5 Hz cut off frequency. Filtered data was subsequently analyzed using the software Acti4, which has been shown to classify physical activity types and postures (e.g. stair climbing, running, walking, standing, moving, sitting and/or lying) with high sensitivity and specificity [19, 20]. Acti4 uses overlapping 2-s intervals to derive instantaneous average parameters (mean and standard deviation of acceleration, inclination) from thigh-worn accelerometer signal. These average parameters are then used to classify physical activity types and postures using the rule-based decision tree described by Skotte et al. 2014 [19]. This classification method has been validated against video analysis and pressure sensors for sitting in free-living setting [20].

### Eligibility criteria

Only participantwith valid days of accelerometer measurements were considered eligible for this study. A valid day consisted of accelerometer data of least 10 waking hours and one measurement of time in bed. Time in bed was based on participants’ diary information and accelerometer-derived periods of ≥4 h in bed at night. These periods were verified by visually comparing accelerometer data (i.e. detection of lying/non-lying activities) and self-reported time going to bed at night and from getting up in the morning. A total of 1310 workers had at least one valid day of accelerometer data with no zeros and were included in the present study.

### Statistical analyses

We compared the performance of three zero replacement methods: 1) simple replacement, 2) multiplicative replacement, and 3) lrEM replacement (see Additional file 2 for detailed descriptions). Briefly, the simple replacement method replaces zeros in each affected behavior with a fixed value and rescales the data to add up to the constant sum. Multiplicative replacement does a similar thing, but in a way that guaranties desirable compositional properties [21]. Specifically, when the multiplicative replacement method is used, the zeros are also replaced by fixed values, but once this is done, the values in the observed (non-zero) behaviors are adjusted multiplicatively in a meaningful way to preserve the ratios between them, i.e. to not modify their relative relationships. These methods are non-parametric and univariate, meaning they do not make any assumption about an underlying probabilistic model for the behavioral variables and they do not use any information about potential relationships between them to impute the zeros. In contrast, the lrEM algorithm is a parametric method (i.e. it relies on a probabilistic model for the data) and, unlike the others, makes use of the information about the co-dependence structure between behaviors to produce estimated values of the zeros. Under the assumed log-ratio multivariate normal model [22], estimates of what to replace zeros with are predicted from compositional censored linear regression (one model per zero pattern in the dataset) of the behavior containing zeros on the behaviors without zeros, through log-ratio representation and taking into account that the estimated value must be lower than a given censoring threshold (i.e. a detection limit or observation threshold).

#### Simulation study

The daily time-use composition used as reference consisted of six parts, expressed in minutes spent sedentary (i.e. sitting, reclining, and lying), standing (including moving, i.e. small movements in an upright posture), walking, running, stair climbing and in bed within a 24-h day. This dataset provided the reference parameters (i.e. the compositional mean and variation matrix, both provided in Additional file 3) for the log-ratio normal model used to simulate samples of accelerometer datasets. We defined a range of six potential scenarios with an increasing number of imposed zeros (from 5 to 30% zeros, in 5% increments). For each of the six scenarios, 1000 random accelerometer datasets were simulated. The zeros were generated in each dataset by setting an artificial observation threshold so that the target percentage of zeros was reached. That is, simulated values falling below the threshold were turned into zeros in accordance with the assumed mechanism that generates rounded zeros in time-use data. This random process generated zeros in either running, stair climbing or both behaviors in all scenarios. Information about the artificial thresholds used to generate the zeros is provided in Additional file 3. The zeros were subsequently replaced using the simple, multiplicative and lrEM methods.

As written above, unlike the lrEM replacement method, the simple and multiplicative replacement methods require the user to choose a fixed value to replace the zeros in the dataset. Given the assumed zero generating mechanism, this should be a value below the threshold used to impose the zeros in each specific scenario. Based on previous simulation experiments, a 65% of the threshold has been recommended to impute when using multiplicative replacement [21]. The performance of each of the replacement methods was assessed by comparing three measures of distortion across the simulated datasets with respect to the complete dataset. Based on a previous study (14), the chosen measures of distortion were: the average difference in geometric means (ADG), the relative difference in total variances (RDTV) and the relative difference in isometric log-ratio (ilr) covariance matrices (RDCM). In short, lower values of ADG, RDTV and RDCM indicate a smaller difference in geometric means, total variance and covariance structure respectively between the datasets. Details about how ADG, RDTV and RDCM were calculated are provided in Additional file 4. The results across simulation runs for each scenario were summarized by the average distortion ± standard deviation (SD) and plotted for comparison.

#### Case study

We produced two example cases, which are likely to occur when analyzing accelerometer data. Using procedures analogous to the simulation study (i.e. increasing the observation threshold so that different percentages of imposed zeros were obtained), we generated two example datasets from the complete dataset by imposing 10 and 20% zeros and replaced these zeros using the simple, multiplicative or lrEM method. Zeros would occur in either running, stair climbing or both behaviors. In the first example case, zeros were replaced using a fixed value of 0.5 min for the simple and multiplicative replacement methods, as previously done within physical behavior time-use epidemiology [23]. Note that this approach, although used in physical activity research, is ignoring the threshold and actually proposing a value over it, which is inconsistent with the zero generating mechanism. In the second example, zeros were replaced using the same procedure as for the simulation study. That is, zeros were replaced with a value corresponding to 65% of the threshold used to impose zeros. Recall that the lrEM method is not affected by these choices because, by construction, it replaces zeros with estimated values below the observation threshold (as written above).

We compared the complete dataset and datasets by using compositional means, which were obtained by computing the geometric mean of each part of the respective composition [4, 5] and then adjusting them to sum up to the same total daily minutes (i.e. 1440 min). We visually compared the effects on the structure of relative variation of the case with 20% zeros through inspection of compositional bi-plots, which are based on principal component analysis (PCA) of the centered log-ratio (clr) transformation of the physical behavior composition [24]. A biplot allows for the simultaneous display of the individuals and behaviors in a two-dimensional coordinate system. The PCA produces an ordered collection of variables (the principal components) partitioning the total variability in the dataset. Under regular conditions, the first two principal components (PC1 and PC2) account for the largest fraction of that total variability and are typically chosen to provide the coordinates of the elements making up the biplot. Specifically, individuals are represented by points (with higher proximity between them suggesting higher similarity between the activity behavior profiles) and behaviors are represented by arrows (with higher proximity between arrowheads indicating stronger co-dependence between behaviors). Thus, the comparison of the resulting biplots allows us to visually assess the distortion introduced by the replacement methods for each of the example datasets. Further details about compositional biplots can be found elsewhere [5, 24].

All replacement methods and analyses were performed in R version 1.1.3 [25], using the *compositions* [26] and *zCompositions* [27] packages. In particular, multiplicative replacement and log-ratio EM algorithm were respectively implemented in the *multRepl* and *lrEM* functions of the *zCompositions* package. For the *lrEM*, we used ordinary maximum likelihood parameter estimates (i.e. the default setting).

## Results

### Study population characteristics

The mean age of the study sample was 45.1 (SD = 10.1) years. Mean BMI was 26.7 (SD = 4.8) and 58% of the sample population were women (Table 1). The majority worked within manufacturing (41%), followed by health services (31%) and cleaning (12%).

**Table 1:** Characteristics of the study sample (*n* = 1310)

Variable	n	%	Mean (SD)	Range
Age in years	1296		45.1 (10.1)	[18.0; 71.0]
BMI in kg/m^2^	1257		26.7 (4.8)	[15.4; 45.1]
Sex				
Men	542	42		
Women	754	58		
Cohort				
NOMAD	230	18		
DPhacto	686	52		
DOSES	394	30		
Working sector				
Cleaning	159	12		
Manufacturing	540	41		
Transportation	73	6		
Health Service	409	31		
Assemblers	33	3		
Construction	39	3		
Garbage Collectors	27	2		
Mobile Plant Operators	10	1		
Other^A^	20	2		

*BMI* body mass index, *SD* standard deviation. ^A^Includes general office clerks and other elementary workers

### Results of simulation study

**Fig. 1:**
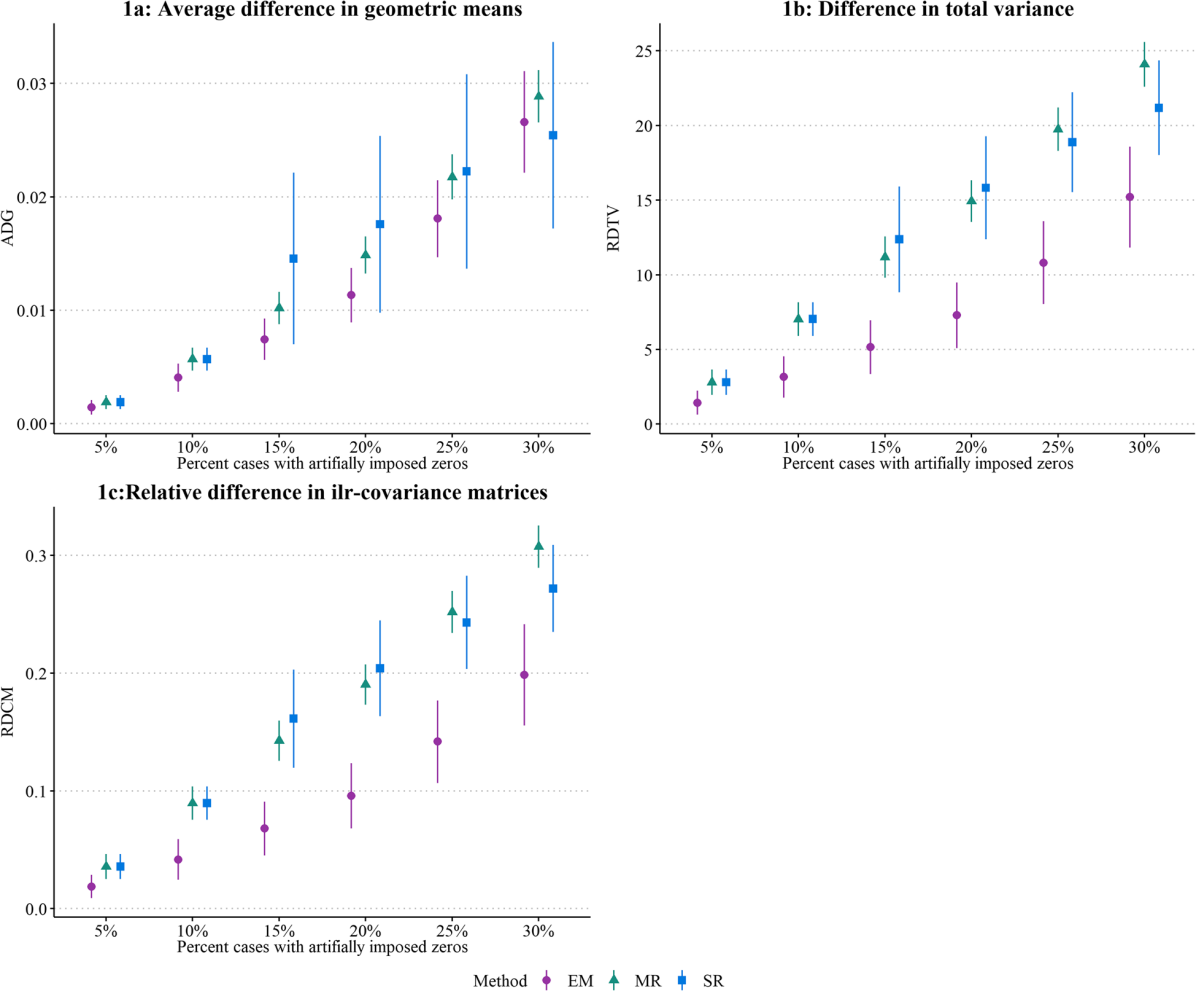
Difference in ADG, RDTV, and RDCM FOR EACH REPLACEMENT METHODS ACROSS THE SIX SIMULATED SCENARIOS. Comparison between complete dataset and dataset with replaced zeros using average difference in geometric means (ADG), relative difference in total variance (RDTV) and relative difference in ilr-covariance matrices (RDCM). Low values for ADG, RDTV and RDCM indicate small differences in geometric means, total variance and covariance structure, respectively, between the complete and replaced datasets. EM is the log-ratio expectation-maximization replacement method, MR is the multiplicative replacement method, and SR is the simple replacement method. The points indicate mean across 1000 simulated data sets and vertical lines represent ± standard deviation

Figure 1 shows the comparison of the replacement methods for each scenario (i.e. from 5 to 30% zeros) by plotting the average values (± SD) across simulations of the three distortion measures (1a: ADG, 1b: RDTV, 1c: RDCM). In all scenarios, the lrEM replacement method showed the smallest distortion in geometric means, total variance, and covariance structure. The simple replacement method showed unstable results, particularly for the ADG and from 10% zeros. The lowest differences in performance between replacement methods were found for the scenario with 5% zeros.

### Results from case study

When comparing the compositional means for the four situations investigated in the case study (i.e., 20 and 10% zeros with different replacement values), the overall differences were small (Table 2).

**Table 2:** Compositional means of complete dataset and datasets with replaced zeros

		Compositional mean in minutes/day					
	Replacement method	SB	Standing	Walking	Running	Stairs	TIB
Complete dataset		516.04	350.54	124.63	1.53	6.14	441.12
20% zeros, Replacement value of 0.5 min^A^	SR	514.01	350.52	124.62	1.62	6.14	441.09
	MR	516.01	350.53	124.62	1.60	6.14	441.10
	lrEM	516.04	350.54	124.63	1.54	6.14	441.12
20% zeros, Considering observation threshold^B^	SR	516.04	350.54	124.63	1.53	6.14	441.12
	MR	516.04	350.54	124.63	1.54	6.14	441.12
	lrEM	516.05	350.54	124.63	1.53	6.14	441.12
10% zeros, Replacement value of 0.5 min^A^	SR	516.03	350.53	124.62	1.58	6.14	441.10
	MR	516.01	350.52	124.62	1.62	6.14	441.09
	lrEM	516.04	350.54	124.63	1.54	6.14	441.12
10% zeros, Considering observation threshold^B^	SR	516.04	350.54	124.63	1.53	6.14	441.12
	MR	516.04	350.54	124.63	1.53	6.14	441.12
	lrEM	516.05	350.54	124.63	1.53	6.14	441.12

*MR* Multiplicative replacement method, *lrEM* log-ratio Expectation-Maximization replacement method, *SB* sedentary behavior, *SR* simple replacement method, *TIB* time in bed. ^A^SR and MR use 0.5 min for replacement, whereas lrEM estimates a value below the observation threshold. ^B^SR and MR use 65% the observation threshold for replacement, whereas lrEM estimates a value below the observation threshold

**Fig. 2:**
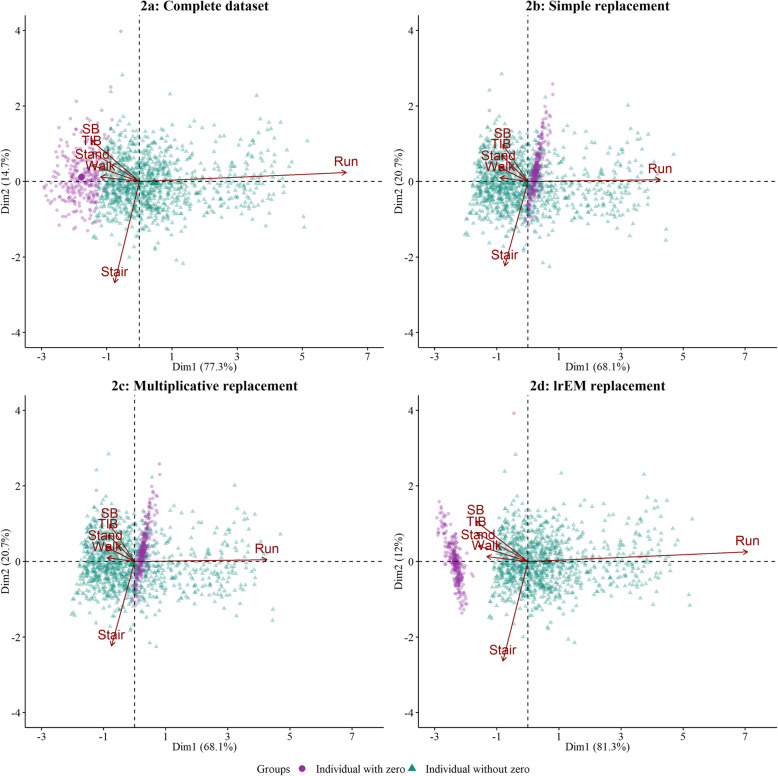
Biplots of complete and replaced datasets (20% zeros, 0.5 minutes replacement). Zeros replaced using simple, multiplicative, and lrEM replacement*.* Note that the use of the fixed value of 0.5 minutes to replace zeros only affects the simple and multiplicative replacement methods, whereas the lrEM method by construction replaces zeros with estimated values below the observation threshold. Individuals for which zeros have been imposed and replaced are indicated with a different color

**Fig. 3:**
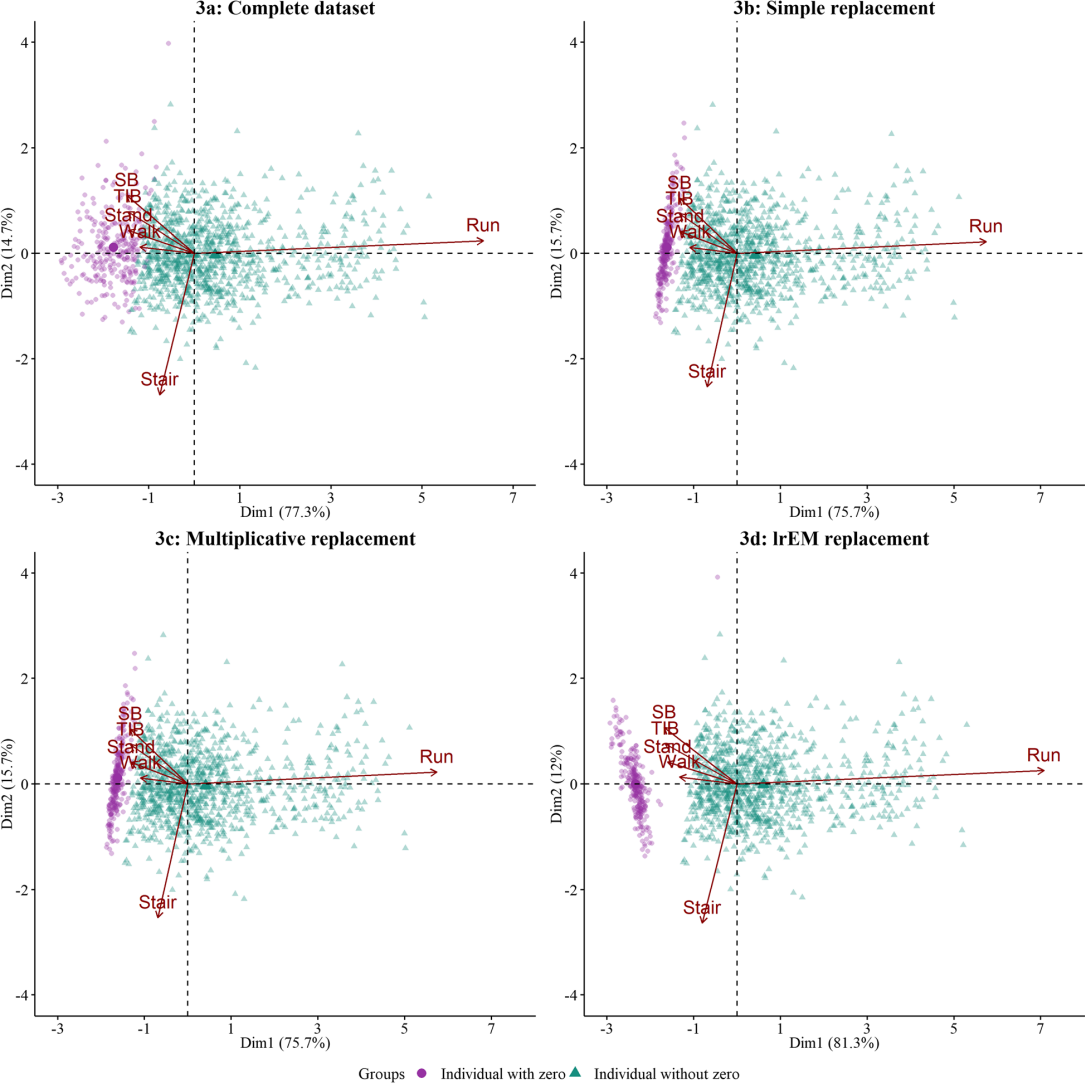
Biplots of complete and replaced datasets (20% zeros, observation threshold-based replacement). Zeros replaced using simple, multiplicative and lrEM replacement. The simple and multiplicative replacement methods were set up to replace zeros with 65% the observation threshold, whereas the lrEM by construction replaces zeros with estimated values below the observation threshold. Individuals with imposed and replaced zeros are indicated with a different color

The compositional biplots in Figs. 2 and 3 illustrate the distortion in the structure of relative variation introduced when using replacement values of 0.5 min or taking into account the observation threshold, respectively.

When using a replacement value of 0.5 min in SR and MR (Fig. 2b and c), individuals for which zeros had been replaced were forcibly translated from their original position at the left end of the cloud of points, as it corresponds for individuals with small values in running (Fig. 2a), to somewhere around the center of the biplot. This means that after zero replacement, individuals with zeros were assigned higher values in running and stair climbing than individuals without zeros in these behaviors. Moreover, compared to the complete dataset (Fig. 2a), we observe a decrease in the distance between the arrowheads associated with running and stair climbing for all replacement methods (Fig. 2b, c, and d). This change indicates an underestimation of the log-ratio variance between the two behaviors (i.e. an overestimation of their co-dependence). These log-ratio variances were 1.98, 1.98 and 2.10 for SR, MR and lrEM respectively, compared with 2.73 for the complete dataset. Finally, the total variability explained by the respectively biplots, these were 88.8, 88.8 and 93.3% for SR, MR and lrEM respectively, in comparison to 92% for the complete dataset (Fig. 2b, c, and d).

We observed improved performance for the SR and the MR, when consideration was taken for the zero generating mechanism and choosing a replacement value considering the observation threshold (Fig. 3). Specifically, for the SR and MR, the distance between the arrowheads associated with running and stair climbing increased slightly, in line with the log-ratio variance between the two behaviors (now 2.55 for both the SR and MR). The total variability explained by the SR and MR biplots also increased to 91.4%.

## Discussion

In this study, we compared the performance of three zero replacement methods using simulated datasets across six different scenarios of zeros (5 to 30%, in 5% increments) in accelerometer data of daily physical behaviors from 1310 adults. Furthermore, we assessed the potential consequences of replacing zeros with values over the observation threshold using datasets with 10 and 20% of imposed zeros. In all simulated scenarios, the lrEM replacement method performed best in preserving the structure of relative variation in the datasets. In contrast, the simple replacement method performed the worst, showing the highest distortion, followed by the multiplicative replacement method, particularly as the proportion of zero values increased. The case study illustrated that replacing zeros with a value over the observation threshold had severe consequences for the structure of the dataset, independent of which replacement method was used.

The poor performance of the simple replacement method is explained by the fact that it replaces zeros with an identical value and it does not take the relative nature of time-use data into account. This failure to take the relative nature into account distorts the ratios between behaviors without zeros [28]. In contrast, the multiplicative replacement method is designed to preserve the ratios between behaviors not involving zeros. Nevertheless, this approach still replaces zeros with identical values for all individuals and can therefore introduce noticeable underestimation of the total variability and exaggerate similarities between behavioral profiles, particularly in cases with more than 10% zeros as found in this study and elsewhere [6, 10, 11, 28]. In contrast, the lrEM method makes use of the structure of co-dependence between behaviors to produce estimates of values to replace the zeros. Accordingly, this method performed the best in maintaining the structure of relative variation of the dataset. Moreover, although technically more sophisticated, lrEM is as straightforward to apply in practice using the *zCompositions* package.

The case study clearly illustrated the negative consequences of using a replacement value, which is not coherent with the zero generating mechanism and artificially set over the observation threshold of the behavior. Specifically, individuals containing zeros, and then assumed to spent very little time on the affected behaviors (i.e. running and stair climbing), appeared to have higher values in these behaviors than individuals without zeros after replacement. Consequently, the distortion in the structure of relative variation of the dataset is further inflated.

That the simple and multiplicative replacement methods distort the structure of relative variation to a notable extent, especially as the incidence of zeros increases in a dataset, is an important finding. Such distortion artificially exaggerates the similarity between individuals. This might lead to misleading results from any subsequent analysis, particularly, when the number of zero values in the dataset is more than 10% [6, 10]. The case study provided further insight into the shortcomings of zero replacement strategies found within physical behavior time-use epidemiology, i.e. replacing zeros with values, which ignore the observation threshold for a behavior. In a real-world setting, this threshold will commonly be a pre-established detection limit (e.g. as provided by the measuring device) or the smallest value observed for the behavior. For compositional data analysis, any replacement method should preserve the ratios between the non-zero parts of the composition [6]. The extent to which zero replacement methods affect the results in a particular study depends on a number of factors. Foremost, it is crucial to consider the likely reason for the zero values and the frequency of their occurrence. However, also the characteristics and size of the study population, the objective of the study, and the statistical methods used and their assumptions will play a role [27]. We encourage researchers to carefully consider the choice of replacement method, whether or not it is the appropriate choice, which biases this choice could introduce, and the potential effect of method on the structure of relative variation of their data. Since it does not appear to be sufficient to judge the effects of a replacement method on the basis of the compositional means of physical behaviors alone, we suggest that an investigation of the effect of replacement method on the structure of relative variation is adopted as a standard analysis step, when dealing with zero values in time-use epidemiology.

### Strengths and limitations of the study

A major strength of this study was the detailed comparison of three replacement methods using several scenarios with different distributions of zeros based on simulated datasets. Moreover, the case study based on real data provided important insight into the distortion introduced in the structure of relative variation when choosing a replacement value, which ignores the information provided by the observation threshold.

In this study, we only compared three replacement methods. These methods were chosen based on what has typically been used for zero replacement for physical behavior data (see appendix A). Nevertheless, it could be considered a limitation that we only compared these select replacement methods, as several other parametric and non-parametric strategies for rounded zero replacement can be found [6, 27].

## Conclusion

We found differences between the investigated replacement methods, particularly in terms of how well they preserved the co-dependence of compositional physical behavior data. Moreover, we observed higher distortion when zeros where replaced with values that did not take the observation threshold for the behaviors into account. Based on our findings, we recommend the use of the lrEM replacement method when replacing moderate amounts of zeros in physical behavior data and a replacement value, which takes into account observation thresholds, either by pre-established limits of detection or the smallest observed values for the behaviors.

## Supplementary information

**Additional file 1:** Results of systematic literature search on zero replacements used within physical behaviours time-use epidemiology.

**Additional file 2:** Description of the simple replacement, multiplicative replacement and lrEM replacement method.

**Additional file 3:** Details on reference dataset and detection limits used in the simulation and case study.

**Additional file 4:** Description of calculation of average difference in geometric means, relative difference in total variance and relative difference in ilr-covariance matrices.

## Data Availability

The datasets analyzed during the current study are available at the Danish National Archives, https://www.sa.dk/en/k/about-us.

## Abbreviations

ADG: Average difference in geometric means
DOSES: Danish Observational Study of Eldercare work and musculoskeletal disorderS cohort
DPhacto: Danish Physical ACTivity cohort with Objective measurements cohort
CoDA: Compositional data analysis
lrEM: Log-ratio expectation-maximisation algorithm
MVPA: Moderate to vigorous physical activity
NOMAD: New Method for Objective Measurements of Physical Activity in Daily Living cohort
PCA: Principal component analysis
RDCM: Relative difference in isometric log-ratio covariance matrices
RDTM: Relative difference in total variances
