# Supplementary material for: Zero problems with compositional data of physical behaviors: a comparison of three zero replacement methods
Source: Int J Behav Nutr Phys Act. 2020 Oct 6;17:126. doi: 10.1186/s12966-020-01029-z (PMC7542467; doi:10.1186/s12966-020-01029-z)
Supplement: Supplementary file 1 — Additional file 1: Results of systematic literature search on zero replacements used within physical behaviours time-use epidemiology. [file 12966_2020_1029_MOESM1_ESM.docx]

**ADDITIONAL FILE 1**

**Search strategy**

A systematic search was performed in January 2020 in the database Pubmed, using the following keywords: “compositional analyses”, “compositional data”, “physical activity”, exercise, sedentary, inactive, sleep*, walking, sitting and standing. No limitations regarding publication date were applied. A secondary search was performed by screening the reference list of each read full-text article.

**Inclusion criteria**

Only studies meeting the following criteria were included in the review: 1) study was in an English-language with full-text available, 2) data were collected among human participants, 3) the study reported which zero replacement method was used if zeros were present in data. Tables below show the search strategy and results of the systematic literature search.

**Table A1.** Search strategy performed in Pubmed the 23^rd^ of January 2020.

| **Theme** | **Search word(s)** | **Results** |
| --- | --- | --- |
| Compositional data | "compositional analyses" | 344 |
|  | ”compositional data” | 521 |
|  | ("compositional data") OR "compositional analyses" | 854 |
| Physical activity, sedentary behaviour and sleep | “Physical activity*” | 111,679 |
|  | Sedentary* | 32,899 |
|  | Exercise[Mesh] OR exercise | 417,611 |
|  | inactive | 105,745 |
|  | sleep* | 214,694 |
|  | walking | 94,622 |
|  | sitting | 21,984 |
|  | standing | 73,948 |
|  | (((((((standing) OR sitting) OR walking) OR sleep*) OR inactive) OR (Exercise[Mesh] OR exercise)) OR Sedentary*) OR “Physical activity*” | 892,117 |
| Search  (23-01-2020) | ((("compositional data") OR "compositional analyses")) AND ((((((((standing) OR sitting) OR walking) OR sleep*) OR inactive) OR (Exercise[Mesh] OR exercise)) OR Sedentary*) OR “Physical activity*”) | 71 |

**Results**

In total, 71 papers were identified through the database searching of which 65 were within physical activity epidemiology. Of these 65 papers, 15 papers reported to have zero values in the dataset and how they were treated (Table A2). Seven used the log-ration EM replacement method, four studies used the simple replacement methods, three studies used the multiplicative replacement method and one study used amalgamation. One study used log-ratio data augmentation algorithm and simple replacement for a sensitivity analyses.

**Table A2.** Studies reporting strategies on handling of zero values in datasets.

| **Reference** | **Year** | **Measurement of physical behaviors** | **Method for handling zeros** | **Activities with zeros** | **Percentages of zeros** | **Value used to replace zeros in case of simple replacement** |
| --- | --- | --- | --- | --- | --- | --- |
| (Powell et al., 2020) | 2020 | Accelerometer. | Simple replacement method. | Moderate-to-vigorous physical activity. | Not reported. | Not specified, authors wrote “very small value”. |
| (Del Pozo Cruz et al., 2020) | 2020 | Accelerometer. | Log-ratio EM replacement method. | Not reported. | Not reported. |  |
| (Ryan et al., 2019) | 2019 | Accelerometer. | Simple replacement method. | Moderate-to-vigorous physical activity. | Not reported. | 0.1, unit not specified. |
| (Rossen, Von Rosen, Johansson, Brismar, & Hagströmer, 2019) | 2019 | Accelerometer. | Log-ratio EM replacement method. | Moderate-to-vigorous physical activity. | 1 % |  |
| (McGregor, Palarea-Albaladejo, Dall, Stamatakis, & Chastin, 2019) | 2019 | Accelerometer. | Log-ratio EM replacement method. | Moderate-to-vigorous physical activity. | Less than 1 % |  |
| (Lund Rasmussen et al., 2019) | 2019 | Accelerometer. | Log-ratio EM replacement method. | Sedentary bouts and active behaviors (i.e. walking, running, stair climbing and biking). |  |  |
| (Hallman, Mathiassen, van der Beek, Jackson, & Coenen, 2019) | 2019 | International Physical Activity Questionnaire (IPAQ). | Simple replacement method. | Standing and walking. | 22 % | 1 min |
| (Foley et al., 2019) | 2019 | Questionnaire. | Combining light and heavy do-it-yourself activities (algamation) and log-ratio EM replacement method. | Light and heavy do-it-yourself activities. | Not reported. |  |
| (Carson et al., 2019) | 2019 | Accelerometer | Simple replacement method. | Vigorous physical activity. | Not reported. | 0.5 minute. |
| (Amagasa et al., 2019) | 2019 | Accelerometer | Log-ratio EM replacement method. | Moderate-to-vigorous physical activity. | Not reported. |  |
| (Olds et al., 2018) | 2018 | Questionnaire (the Multimedia Activity Recall for Children and Adults (MARCA)) | Multiplicative replacement method | Not reported. | Not reported. |  |
| (Hunt, Williams, Olds, & Dumuid, 2018) | 2018 | Questionnaire (the Multimedia Activity Recall for Children and Adults (MARCA)) and accelerometer | Multiplicative replacement method | Not reported. | Not reported. |  |
| (Gupta et al., 2018) | 2018 | Accelerometer. | Combining physical activities (algamation) | Running, cycling and stair climbing | Not reported. |  |
| (Foley, Dumuid, Atkin, Olds, & Ogilvie, 2018) | 2018 | Diary (starting at 4 am and covering a full 24 hour day, in 10-min timeslots). | Log-ratio EM replacement method and sensitivity analyses for which one minute was imputed for all zero values. | Not reported. | Not reported. |  |
| (Wong et al., 2017) | 2017 | Questionnaire (the Multimedia Activity Recall for Children and Adults (MARCA)). | Multiplicative replacement method. | Not reported. | Not reported. |  |
