# Supplementary material for: Zero problems with compositional data of physical behaviors: a comparison of three zero replacement methods
Source: Int J Behav Nutr Phys Act. 2020 Oct 6;17:126. doi: 10.1186/s12966-020-01029-z (PMC7542467; doi:10.1186/s12966-020-01029-z)
Supplement: Supplementary file 2 — Additional file 2: Description of the simple replacement, multiplicative replacement and lrEM replacement method. [file 12966_2020_1029_MOESM2_ESM.docx]

**ADDITIONAL FILE 2**

**Simple replacement**

The simple replacement method consists of replacing the zeros by a small value and then rescaling the parts of the composition to add up to the corresponding constant sum [1]. If we consider a composition$\boldsymbol{X}$ of *D* parts, such that $\boldsymbol{X}=[x_{1}\ldots x_{D}]$, where $\boldsymbol{X}$ contains rounded zeros, the simple replacement method will impute $\boldsymbol{X}$ by a new composition without zeros$, \boldsymbol{R}=[r_{1}\ldots r_{D}]$, according to the following replacement equation:

| $r_{j}=\left\{ \begin{aligned} \frac{c}{c+\sum_{k\vert x_{k}=0} \delta_{k}}\delta_{j}, if x_{j}=0 \\ \frac{c}{c+\sum_{k\vert x_{k}=0} \delta_{k}}x_{j}, if x_{j}>0 \end{aligned} \right.$ | (1) |
| --- | --- |

where $\delta$ stands for a fixed arbitrary small value which is freely chosen and *c* is the constant sum considered (e.g. 1440 if daily minutes). In the simulation study, $\delta$ was set as the same observation threshold used to simulate the zeros. In the real data case study, we chose a value of 0.5 minutes for $\delta$.

**Multiplicative replacement**

If we consider a composition$\boldsymbol{X}$ with *D* parts, such that $\boldsymbol{X}=[x_{1}\ldots x_{D}]$; where $\boldsymbol{X}$ contains rounded zeros. The multiplicative replacement method [2] will impute $\boldsymbol{X}$ by a new composition without zeros$, \boldsymbol{R}=[r_{1}\ldots r_{D}]$, according to the following replacement equation:

| $r_{j}=\left\{ \begin{aligned} \delta_{j}, if x_{j}=0 \\ \left( 1-\frac{\sum_{k\vert x_{k}=0} \delta_{k}}{c} \right)x_{j}, if x_{j}>0 \end{aligned} \right.$ | (2) |
| --- | --- |

where $\delta_{j}$ is the imputed value on the compositional part $x_{j}$ and *c* is the constant sum constraint. Again, the value of $\delta$ can be freely chosen. However, it is suggested that when $\delta$ is close to 65 % of the detection limit (observation threshold) the distortion of the covariance structure of the dataset is minimized [2], assuming that the proportion of rounded zeros in the dataset is small (i.e. < 10 %). In the simulation study, $\delta$ was set as the same observation threshold used to simulate the zeros. To facilitate comparability with the simple replacement method, we chose to set $\delta$ to 0.5 minutes when performing the multiplicative replacement for real data case study.

**Log-ratio EM replacement**

This method was introduced in [3]. In short, zero values are imputed by small values based on the information provided by the observed data while accounting for a given censoring threshold. Again, we consider a composition $\boldsymbol{X}=[x_{1}\ldots x_{D}]$, which includes rounded zeros. Initially, the compositional dataset $\boldsymbol{X}$ is transformed using either additive or isometric log-ratio transformation (they both provide the same results) into a real dataset $\boldsymbol{Y}=[y_{1}\ldots y_{D}]$. In brief (see [3] for more details), an iterative process is initiated consisting of two steps:

1. E-step: at iteration $t$, given parameter estimates $\hat{\theta}^{(t)}$ of a multivariate normal distribution for the complete dataset, values for the variables containing zeros (assumed to be non-observed small values) in a zero pattern are imputed from the values observed in the other variables fitting a regression equation of the form

| ${{\hat{\boldsymbol{y}}}_{non}}^{(t)}\boldsymbol{=}\boldsymbol{y}_{obs}\hat{\beta}^{(t)}\boldsymbol{-}\hat{\sigma}^{\left( t \right)}\hat{\lambda}^{\left( t \right)}$**,** with $\hat{\lambda}^{\left( t \right)}=\frac{\phi\left( \left( \psi-\boldsymbol{y}_{obs}\hat{\beta}^{(t)} \right)/\hat{\sigma}^{\left( t \right)} \right)}{\Phi\left( \left( \psi-\boldsymbol{y}_{obs}\hat{\beta}^{(t)} \right)/\hat{\sigma}^{\left( t \right)} \right)}$, | (3) |
| --- | --- |

where $\hat{\beta}^{(t)}$ and $\hat{\sigma}^{\left( t \right)}$ stand for maximum likelihood estimates of the regression parameters and the conditional variance respectively. The second term involves the density function, $\phi$, and the distribution function, $\Phi$, of the standard normal model. This term accounts for the observation threshold used ($\psi$ is the log-ratio transformed observation threshold), so that equation (3) always produces values below it once the data are back-transformed into compositions.

1. M-step: an updated estimate $\hat{\theta}^{(t+1)}$ is obtained from the newly imputed dataset and this is used as input for a new E-step.

From an initial estimation $\hat{\theta}^{(0)}$, the E- and M-step are repeated alternatively, generating a sequence of $\hat{\theta}^{(t)}$ estimates until the difference between two consecutive ones is sufficiently small. Once convergence has been reached, the last imputed dataset is transformed back through inverse log-ratio transformation to obtain the corresponding imputed compositional dataset without rounded zeros.

**REFERENCES**

1. Aitchson J. The statistical analysis of compositional data. Chapman and Hall, London; 1986.

2. Martín-Fernández JA, Barceló-Vidal C, Pawlowsky-Glahn V. Dealing with Zeros and Missing Values in Compositional Data Sets Using Nonparametric Imputation. Mathematical Geology. 2003;35:253–78.

3. Palarea-Albaladejo J, Martín-Fernández JA, Gómez-García J. A Parametric Approach for Dealing with Compositional Rounded Zeros. Math Geol. 2007;39:625–45.
