# Supplementary material for: Zero problems with compositional data of physical behaviors: a comparison of three zero replacement methods
Source: Int J Behav Nutr Phys Act. 2020 Oct 6;17:126. doi: 10.1186/s12966-020-01029-z (PMC7542467; doi:10.1186/s12966-020-01029-z)
Supplement: Supplementary file 3 — Additional file 3: Details on reference dataset and detection limits used in the simulation and case study. [file 12966_2020_1029_MOESM3_ESM.docx]

**ADDITIONAL FILE 3**

Table C1 shows the compositional mean (expressed in percentages) and variation matrix from the reference accelerometer dataset used as parameters of the log-ratio normal model in the simulation study.

The six detection limits (expressed in percentages) used to reach the target percentage of zeros (from 5 to 30%) in the scenarios set up for the simulation study are provided in table C2.

The three detection limits (expressed in minutes) used to reach the target percentage of zeros in the case study based on real data are provided in table C3.

**Table C1.** Compositional mean (CM), expressed in percentages, and variation matrix for the reference dataset.

|  | **SB** | **Standing** | **Walking** | **Running** | **Stairs** | **TIB** |
| --- | --- | --- | --- | --- | --- | --- |
| CM (in %) | 35.84 | 24.34 | 8.65 | 0.11 | 0.43 | 30.63 |
| *Variation matrix* | | | | | | |
| SB | 0 |  |  |  |  |  |
| Standing | 0.28 | 0 |  |  |  |  |
| Walking | 0.26 | 0.11 | 0 |  |  |  |
| Running | 2.94 | 2.87 | 2.69 | 0 |  |  |
| Stairs | 0.73 | 0.57 | 0.47 | 2.73 | 0 |  |
| TIB | 0.10 | 0.12 | 0.14 | 2.85 | 0.60 | 0 |

*CM=compositional mean. SB=sedentary behavior (sitting and lying). TIB = time in bed.*

**Table C2.** Detection limits (DL) used to reach target percentage of zeros (indicated in parenthesis) in each scenario of the simulation study and actual percentage of zeros obtained (mean percentage and standard deviation across 1000 simulated data sets per scenario).

| **Scenario #** | **DL** | **Mean percentage of zeros (SD)** |
| --- | --- | --- |
| 1 (5%) | 0.00001 | 4.7 (0.6) |
| 2 (10%) | 0.00002 | 10.2 (0.8) |
| 3 (15%) | 0.00003 | 15.2 (0.1) |
| 4 (20%) | 0.00004 | 19.6 (1.1) |
| 5 (25%) | 0.00005 | 25.07 (1.1) |
| 6 (30%) | 0.00007 | 30.03 (1.3) |

*DL=Detection limit. SD=standard deviation.*

**Table C3.** Detection limits (DL) used to reach target percentage of zeros in case study.

| **DL** | **Percentage of zeros** |
| --- | --- |
| 0.097 | 30 % |
| 0.063 | 20 % |
| 0.033 | 10 % |

*DL=Detection limit.*
