# Supplementary material for: Zero problems with compositional data of physical behaviors: a comparison of three zero replacement methods
Source: Int J Behav Nutr Phys Act. 2020 Oct 6;17:126. doi: 10.1186/s12966-020-01029-z (PMC7542467; doi:10.1186/s12966-020-01029-z)
Supplement: Supplementary file 4 — Additional file 4: Description of calculation of average difference in geometric means, relative difference in total variance and relative difference in ilr-covariance matrices. [file 12966_2020_1029_MOESM4_ESM.docx]

**ADDITIONAL FILE 4**

In the following, we refer to the complete dataset (i.e. compositional data without zeros) as $\boldsymbol{X}$ and the imputed dataset as $\boldsymbol{X}^{*}$. The formulas used are based on (15).

**Average difference in geometric means (ADG)**

The ADG is calculated as:

| $ADG=\frac{d_{a}(g\left( \boldsymbol{X} \right), g\left( \boldsymbol{X}^{*} \right))}{D}*100,$ | (1) |
| --- | --- |

where $g\left( \boldsymbol{\cdot} \right)$ refers to the geometric mean of the original ***X*** and imputed $\boldsymbol{X}^{*}$ datasets. The $d_{a}$ denotes the Aitchison distance (see e.g. (6)), which is used to measure the difference between them. A lower value of ADG indicates a smaller difference in geometric means between datasets.

**Relative difference in total variance (RDTV)**

The relative difference in total variance is calculated as:

| $RDTV= \frac{totvar\left( \boldsymbol{X}^{*} \right)-totvar\left( \boldsymbol{X} \right)}{totvar\left( \boldsymbol{X} \right)}*100,$  with$totvar\left( \boldsymbol{X} \right)=\frac{1}{n}\sum_{i=1}^{n} d_{a}^{2}(\boldsymbol{x}_{i},g(\boldsymbol{X}))$, | (2) |
| --- | --- |

where $g\left( \boldsymbol{\cdot} \right)$ and $d_{a}$ denote the geometric mean and Aitchison distance respectively and $n$ is the number of individuals in the dataset. A lower value of RDTV indicates a smaller difference in total variance between the datasets with respect to the complete dataset.

**Relative difference in ilr-covariance matrices (RDCM)**

The RDCM is calculated as:

| $RDVM=\frac{\left\Vert\boldsymbol{S}^{\boldsymbol{*}}\boldsymbol{-S} \right\Vert_{F}}{\left\Vert\boldsymbol{S} \right\Vert_{F}}$ , | (3) |
| --- | --- |

where $\left\| \boldsymbol{S} \right\|_{F}$ and $\left\| \boldsymbol{S}^{\boldsymbol{*}} \right\|_{F}$ denote the Frobenius matrix norm of the covariance matrix in ilr-coordinates (i.e. $\boldsymbol{S}$ and $\boldsymbol{S}^{\boldsymbol{*}}$) of the complete and imputed datasets, respectively. Hence, RDCM serves as a measure of the distortion in the co-dependence structure, with lower values of RDVM indicating smaller distortion with respect to the complete dataset.
